# Supplementary material for: Oleuropein Ameliorates Bleomycin-Induced Pulmonary Fibrosis in Mice by Targeting TGF-β1 Signaling Pathway
Source: Biomolecules. 2025 Aug 22;15(9):1211. doi: 10.3390/biom15091211 (PMC12467347; doi:10.3390/biom15091211)
Supplement: Supplementary file 1 [file biomolecules-15-01211-s001.zip › biomolecules-3733046-supplementary.pdf]

Figure S1:

Original gels of Figure 3

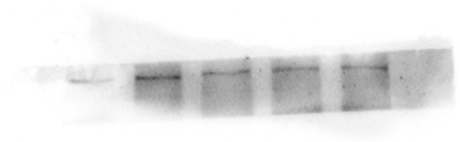

collagen I

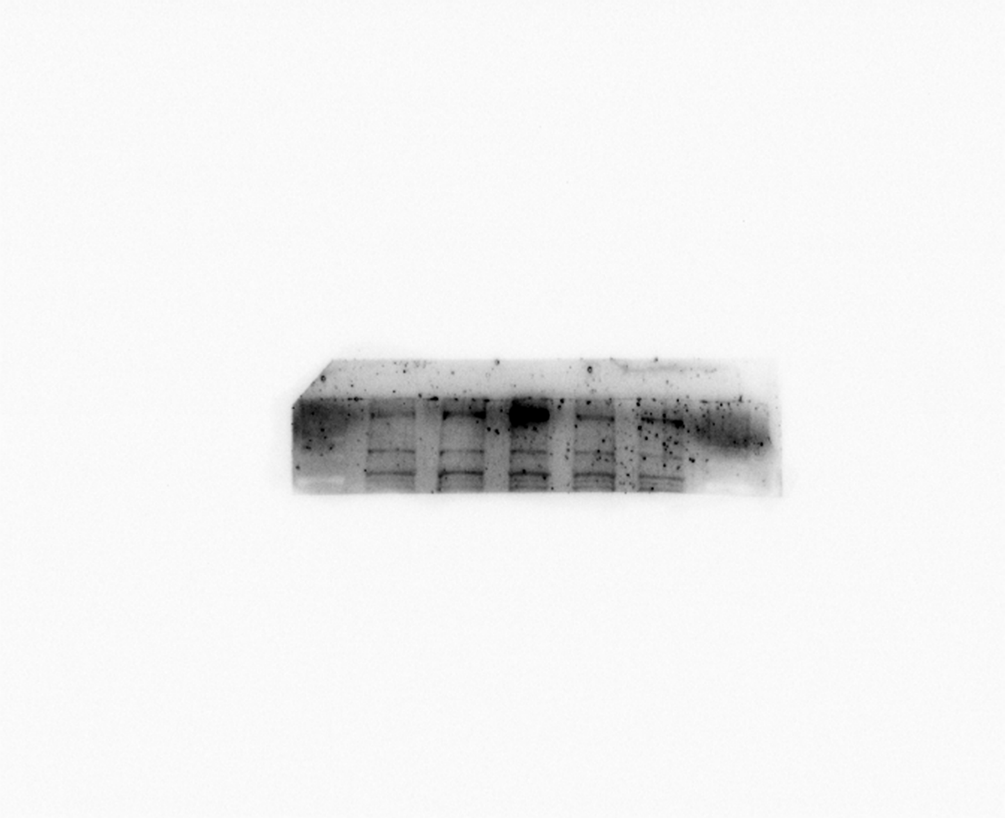

Fibronectin

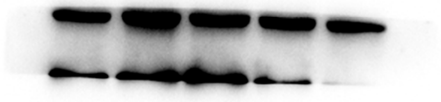

α-SMA

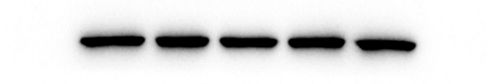

β-tubulin

Original gels of Figure 4

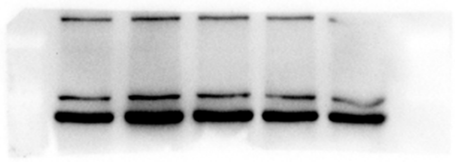

p-Smad2

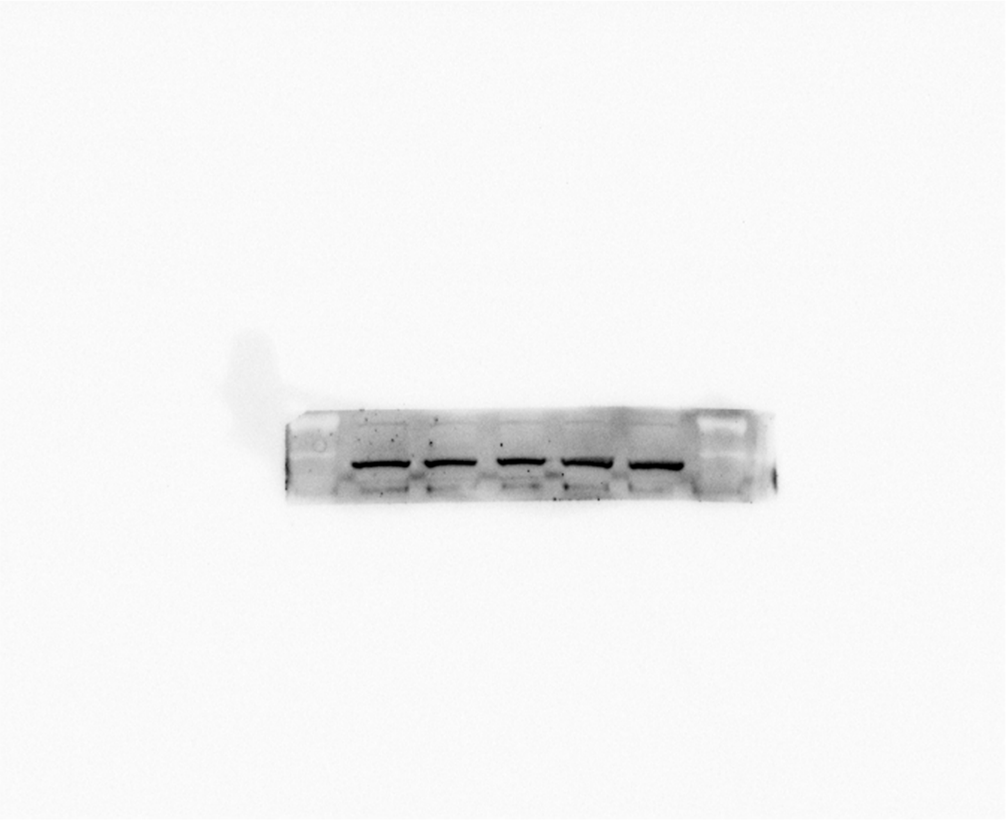

Smad2

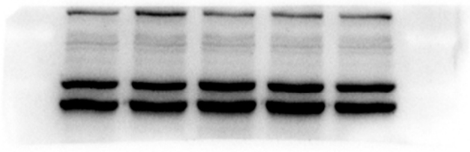

p-Smad3

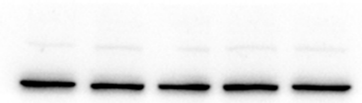

Smad3

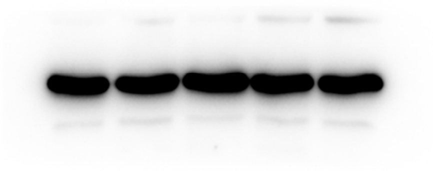

GAPDH

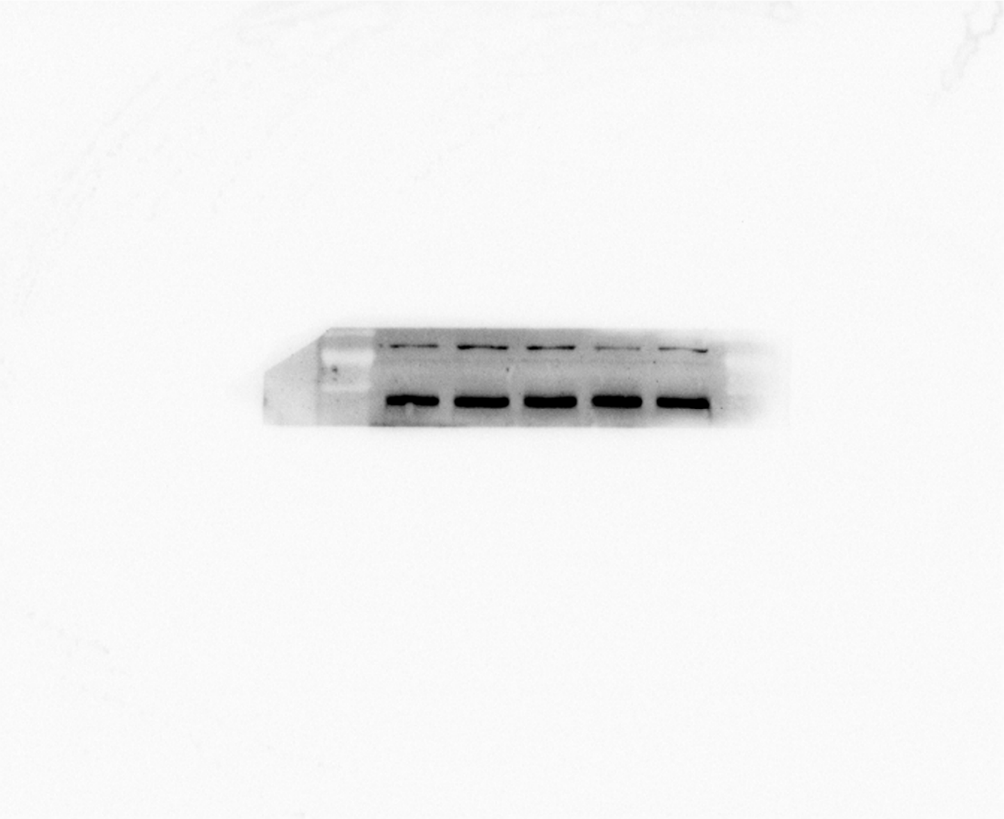

p-Smad2

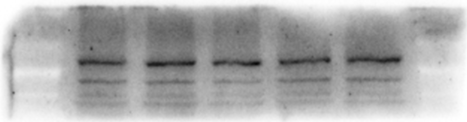

Smad2

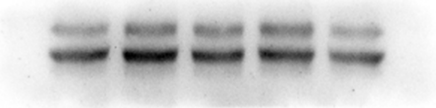

p-Smad3

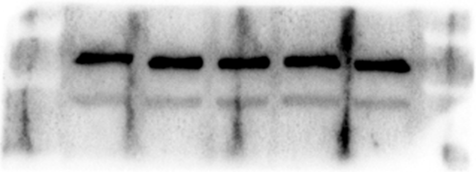

Smad3

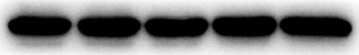

GAPDH

Original gels of Figure 5

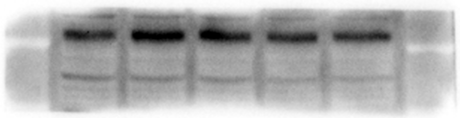

p62

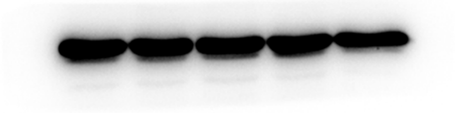

GAPDH

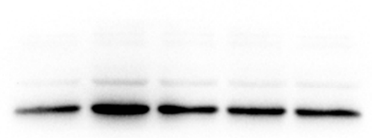

p62

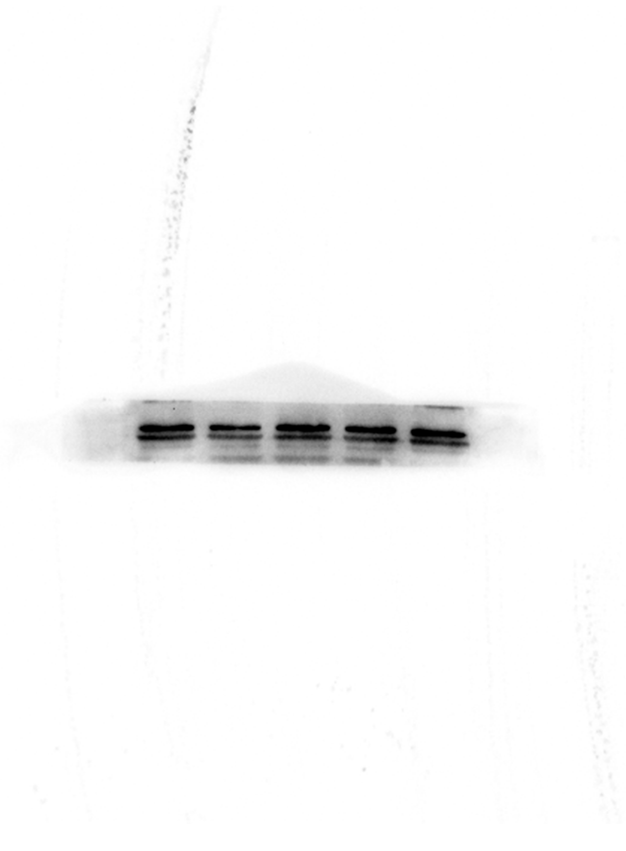

Beclin 1

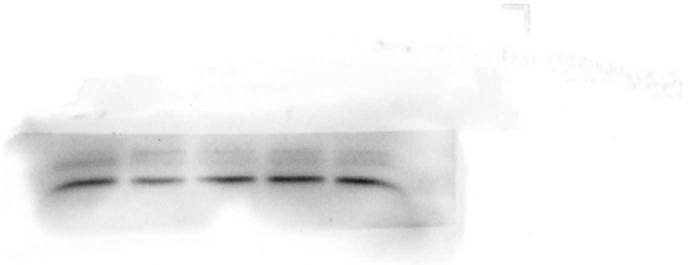

LC3-I/II

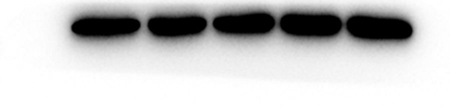

GAPDH

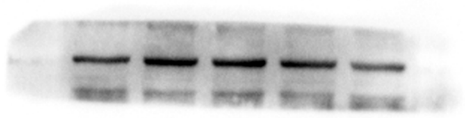

p62

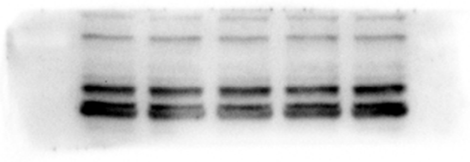

Beclin 1

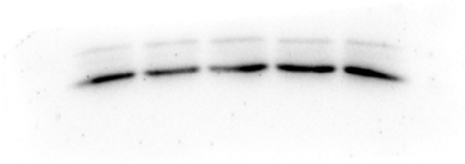

LC3-I/II

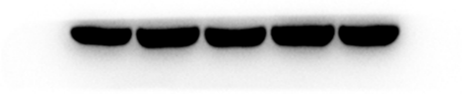

β-actin

Original gels of Figure 6

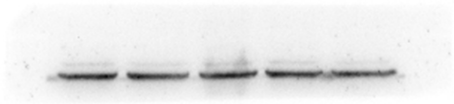

Caspase9

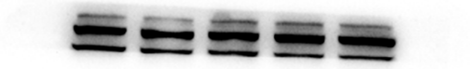

Cleaved-Caspase9

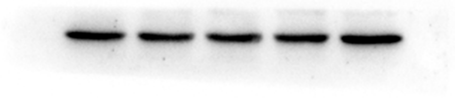

Caspase3

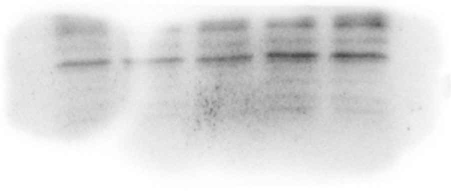

Cleaved-Caspase3

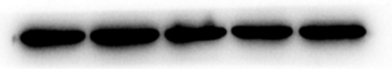

GAPDH

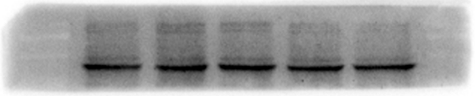

Caspase9

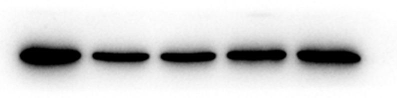

Cleaved-Caspase9

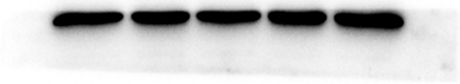

Caspase3

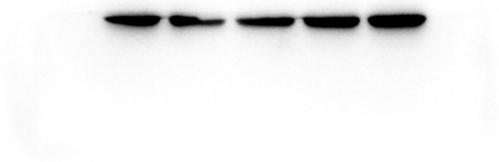

Cleaved-Caspase3

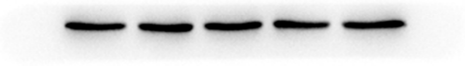

GAPDH

Original gels of Figure 7

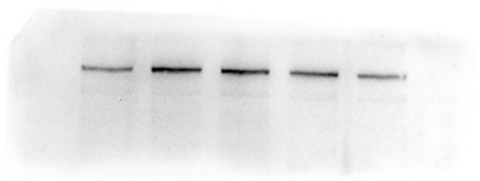

p-mTOR

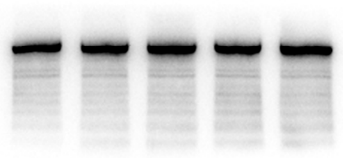

mTOR

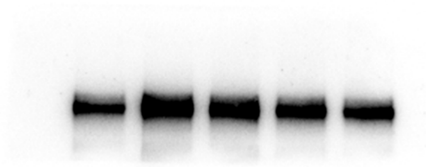

p-ULK1

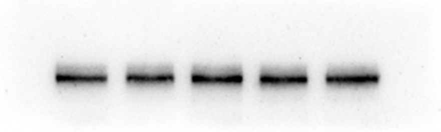

ULK1

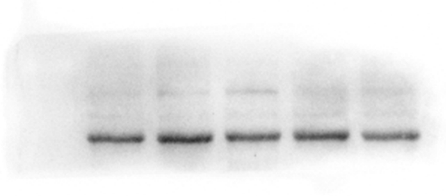

p-p70 S6K

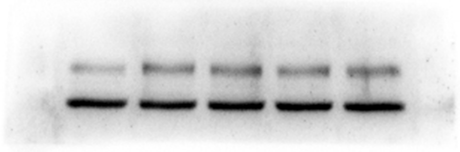

p70 S6K

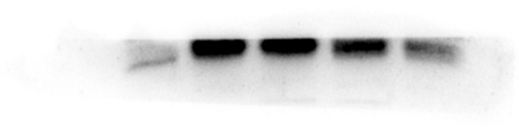

p-S6RP

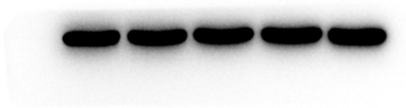

S6RP

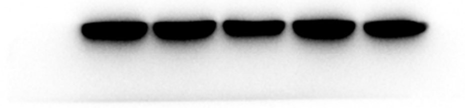

β-actin

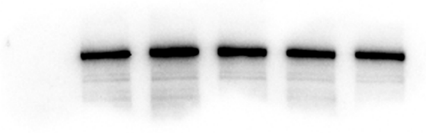

p-mTOR

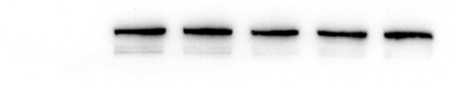

mTOR

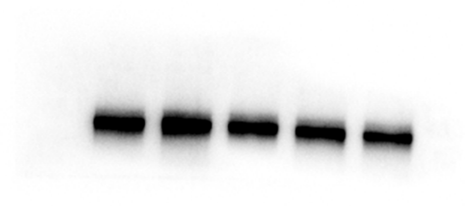

p-ULK1

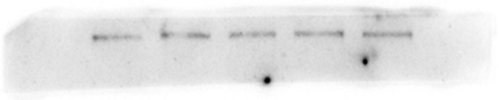

ULK1

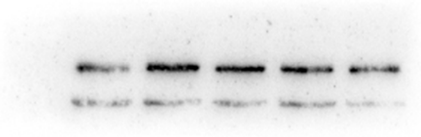

p-p70 S6K

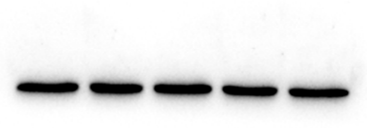

p70 S6K

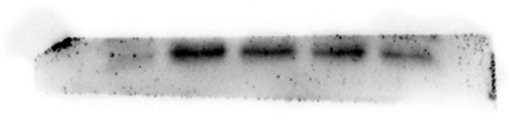

p-S6RP

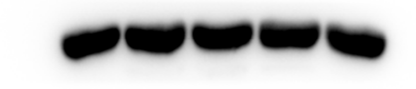

S6RP

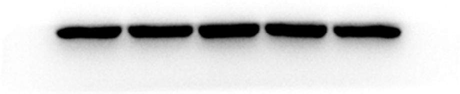

β-actin

Original gels of Figure 8

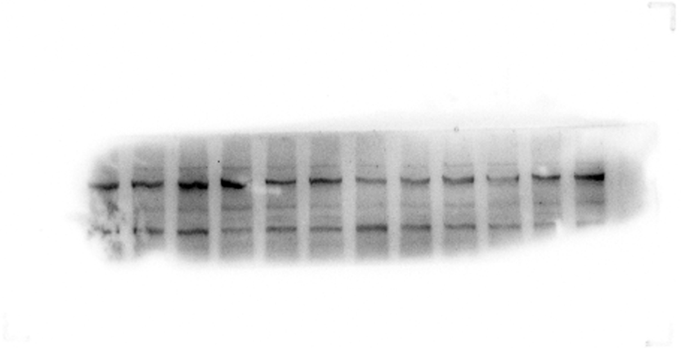

collagen I

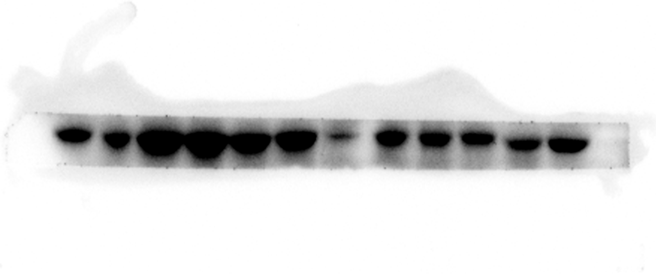

α-SMA

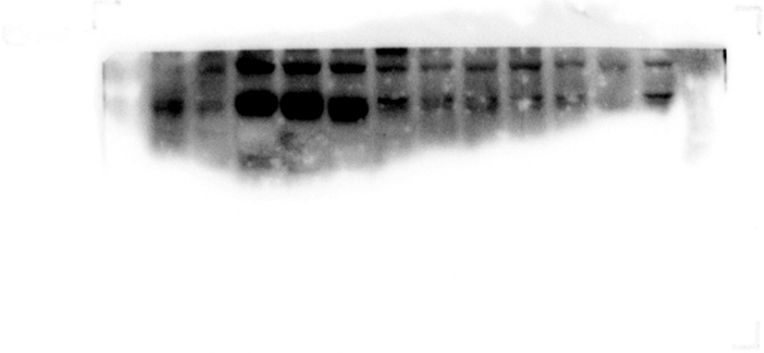

p62

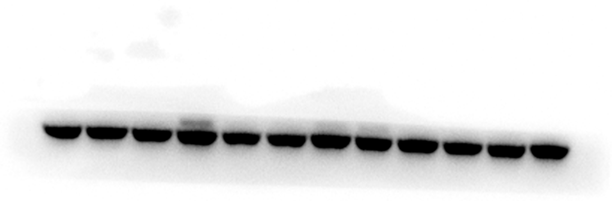

GAPDH

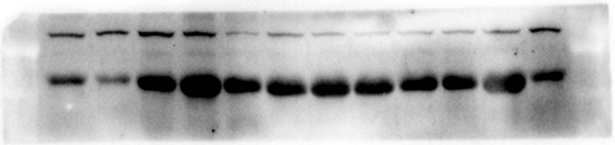

p-Smad2

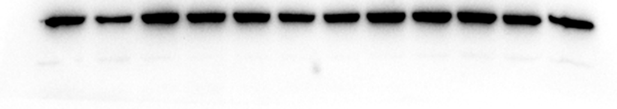

Smad2

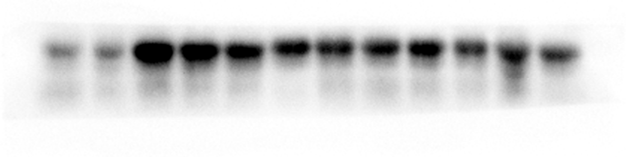

p-Smad3

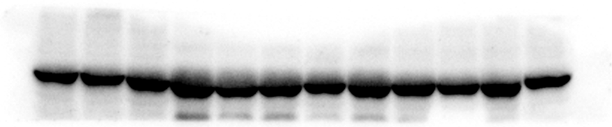

Smad3

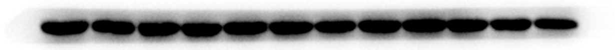

GAPDH

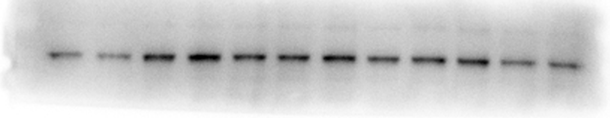

p-mTOR

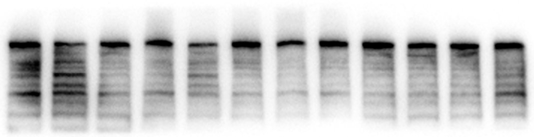

mTOR

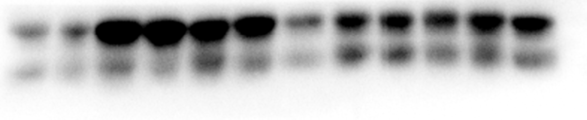

p-S6RP

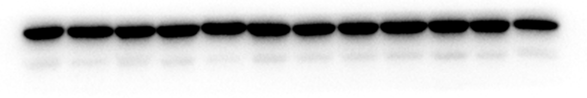

S6RP

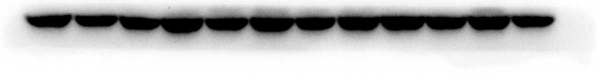

β-actin

Original gels of Figure 9

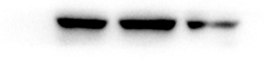

FAP-α

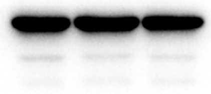

β-tubulin

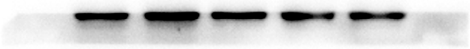

α-SMA

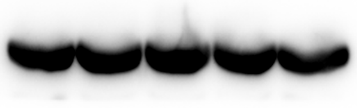

GAPDH

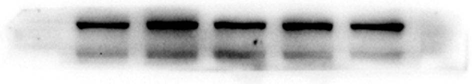

P-Smad3

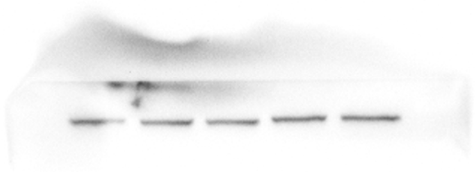

Smad3

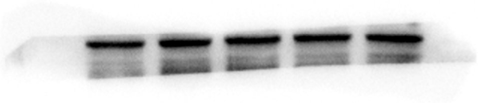

β-tubulin

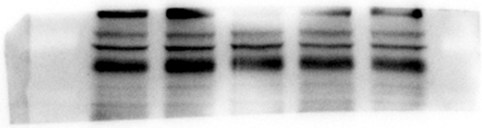

P62

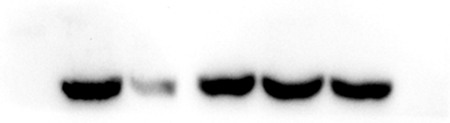

Cleaved-Caspase9

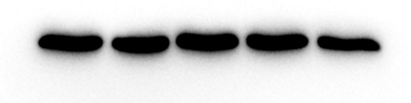

Caspase9

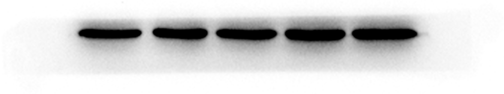

β-tubulin

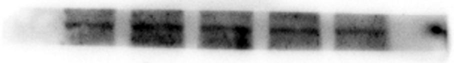

P-mTOR

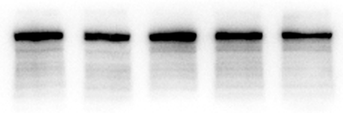

mTOR

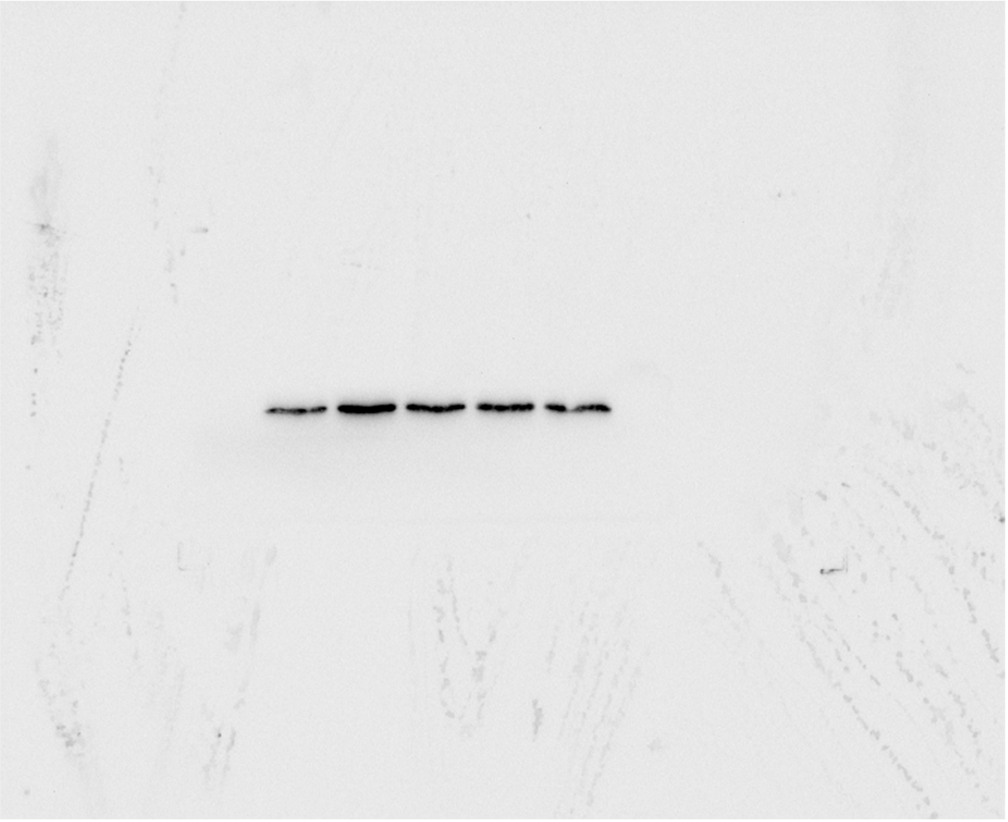

P-S6RP

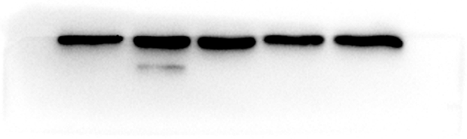

S6RP

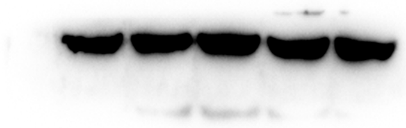

GAPDH
